# Small-Sized Tomato Pomace: Source of Bioactive Compounds and Ingredient for Sustainable Production of Functional Bread

**DOI:** 10.3390/foods13213492

**Published:** 2024-10-31

**Authors:** Selina Brighina, Luana Pulvirenti, Laura Siracusa, Elena Arena, Maria Veronica Faulisi, Cristina Restuccia

**Affiliations:** 1 Dipartimento di Agricoltura, Alimentazione e Ambiente (Di3A), University of Catania, Via S. Sofia 100, 95123 Catania, Italy; selina.brighina@unict.it (S.B.); faulisiveronica4@gmail.com (M.V.F.); crestu@unict.it (C.R.); 2 Consiglio Nazionale delle Ricerche-Istituto di Chimica Biomolecolare (CNR-ICB), Via Paolo Gaifami 18, 95126 Catania, Italy; l.pulvirenti@icb.cnr.it (L.P.); laura.siracusa@icb.cnr.it (L.S.)

**Keywords:** tomato processing waste, polyphenols, antioxidant activity, fibre, carotenoids, sensory profile

## Abstract

Tomato processing generates a by-product known as tomato pomace (TP), which contains chemically diverse valuable components such as lycopene, phenols, dietary fibre, proteins, and oil. The aim of this study was to characterize bioactive compounds in small-sized tomato pomace from cherry and date tomatoes and to evaluate the effects of the addition of 10 and 20% (*w*/*w*) of tomato pomace flour (TPF) to durum wheat dough for bread production. Bread containing different amounts of TPF was characterized by physical, chemical, nutritional, and sensory characteristics. TPF is an important source of dietary fibre with a total content of approximately 52.3%, of which 5.3% is soluble and 47% is insoluble. It is also a potential source of natural antioxidants and contains remarkable residual levels of both total carotenoids and polyphenols. TPF addition reduced water loss during baking and significantly affected colour parameters and acidity; furthermore, both fortified TPF breads could use these nutrition and health claims and label the breads as “High Fibre”. The “overall” sensory attribute showed similar values in the control and fortified bread samples, suggesting that the overall quality of the bread remained relatively constant, regardless of the percentage of added TPF.

## 1. Introduction

Currently, food is intended to offer health benefits that extend beyond its nutritional value, and consumer demand for functional foods has steadily increased.

With reference to the bread-making sector, in recent years, scientific research has focused, on one hand, on reducing sodium intake [1,2,3] and on the other hand, on producing bread enriched in fibre, protein, and/or bioactive compounds [4,5,6,7,8,9]. Tomato (*Lycopersicon esculentum* Mill.) is one of the most widely cultivated vegetable crops in Mediterranean countries. Significant amounts are consumed in the form of processed products such as tomato juice, paste, ketchup, sauce, and salsa. The report published by Ismea [10] confirms that Italy has a fundamental role in world production, being the third producer of fresh tomatoes destined for processing (15% of world production); it is also confirmed as the leading producer and exporter of tomato derivatives. Tomato processing cycles generate a by-product, known as tomato pomace (TP), representing, at most, 4% of the fresh fruit weight; this percentage increases when small-sized tomatoes such as cherry and date tomatoes are processed since they have a relatively high peel-to-volume ratio. Industrially derived TP is generally made up of a mixture of tomato peels and seeds, whose chemical composition is distinctly different from each other; on the whole, tomato pomace contains chemically diverse and valuable components such as lycopene, phenols, dietary fibre, proteins, and oil [11,12,13,14], which are reported to possess anti-inflammatory, antioxidant, and chemopreventive properties [15]. Furthermore, tomato peels were found to contain high levels of potassium ions and relatively low amounts of sodium; this makes the Na/K ratio lower, and makes tomato peels a potentially useful supplement for people suffering from cardiovascular diseases [13].

Thus, considering the potential health benefits of tomato pomace, which is usually used as a feed additive, and the increasing consumer trend towards functional/fortified foods, several attempts for its incorporation into different food matrices have been made [13,16,17,18,19].

Partial replacement of refined flour with tomato pomace in bread, muffins, cookies, cream crackers, and pasta increases the levels of minerals (Ca, Fe, Na, Mg, K, Mn, Zn), fibre, vitamin C, carotenoids, polyphenols, antioxidant activity, and the darker colour of the products [16,18,19,20]. The use of tomato pomace influences the rheological properties of the dough, increasing the level of tomato seed flour, and inducing a decrease in dough extensibility, swelling index, and backing strength, as well as loaf volume, porosity, and elasticity of the bread samples [21]. In cookies and cream crackers, the incorporation of tomato pomace increased the spread factor and the replacement of wheat flour with tomato pomace powder (up to 5% and 8%, for cookies and cream crackers) was found acceptable by the panellists [19,22]. Whilst tomato pomace addition undoubtedly improved the nutrient content of both pasta and gluten-free pasta [17,23], lower sensory scores for elasticity, odour, and firmness were recorded, which indicates the necessity for the addition of hydrocolloids [23].

Moreover, the use of tomato pomace as an ingredient perfectly fits into the “Farm to Fork” (F2F) European Commission strategy, which aims to halve food waste by 2030 and ensure sustainable food production.

To the best of our knowledge, no study has focused on the possible use of tomato pomace from “small-sized” tomatoes, such as cherry and date tomatoes, as an ingredient in the preparation of durum wheat bread. With this information considered, the aims of the present study were (i) to characterize bioactive compounds of small-sized tomato pomace obtained by different food-grade extractions; (ii) to evaluate the effects of the addition of different amounts of tomato pomace on the durum wheat dough for bread production; and (iii) to characterize the functional bread obtained by addition of different amounts of tomato pomace for physical, chemical, and nutritional parameters, and sensory characteristics.

## 2. Materials and Methods

### 2.1. Preparation of Tomato Pomace Flour (TPF)

Tomato pomace (TP) (a mixture of peels, seeds, and residual pulp in small amounts) was industrially obtained from the processing of small tomatoes (cherry and date tomatoes) and stored at −20 °C. To obtain a homogeneous matrix as an ingredient for durum wheat bread, TP was placed in a circulating air oven (ThermoScientific, Heratherm oven, Waltham, MA, USA) at 80 °C overnight, then finely ground using a blender (La Moulinette, Moulinex, Écully, France) and passed through a sieve with a mesh size of 500 µm. The tomato pomace flour (TPF) obtained was characterized for moisture, lipid content, and fatty acid profiles, fibre carotenoids, total polyphenol content and profile, and antioxidant activity. All the analyses were performed in triplicate.

#### 2.1.1. TPF Characterization

Moisture content was determined by drying at 105 °C until constant weight with a circulating air oven (ThermoScientific, Heratherm oven, MA, USA).

Lipid content was estimated according to the acid hydrolysis method [24]. Five grams of the ground sample was placed with 2 mL of ethanol (95%) and 10 mL of HCl solution (25 mL HCl 37% + 110 mL distilled water) in a glass flask and heated at 70–80 °C for 30 min with stirring. After cooling, 10 mL of ethanol (95%) was added, and the sample was transferred to a separatory funnel. The glass flask was washed with 25 mL of ethyl ether; after, 25 mL of petroleum ether was added, and the mixture was stirred. The extraction of total lipids was repeated twice with 25 mL of ethyl and petroleum ethers (1:1). After separation, the ether extracts were filtered on anhydrous Na_2_SO_4_ and evaporated under a vacuum. The lipid residue was dried in a 100 °C oven (ThermoScientific, Heratherm oven, Waltham, MA, USA) to constant weight.

Total (TDF), soluble (SDF), and insoluble (IDF) dietary fibre was determined according to the enzymatic method AOAC 991.43 [24] using the Total Dietary Fiber Assay Kit (K-TDFR, Megazyme, Ireland).

Carotenoid determination was conducted according to Jaeger de Carvalho et al. [25]. A total of 15 g of the sample was extracted with 25 mL of acetone and filtered under a vacuum. The extraction was conducted three or more times until the TP became colourless. Successively, the acetone extract was transferred in a separatory funnel, an aliquot of petroleum ether was added, and the acetone was removed by the addition of distilled water. The water was discarded, and the procedure was repeated four times. The ether fraction was passed on anhydrous sodium sulphate and transferred in a volumetric flask, and the volume was made up of petroleum ether. The absorbance was read at 450 nm, using a Perkin Elmer lambda 25 UV-VIS spectrometer (PerkinElmer Inc, Waltham, MA, USA).

#### 2.1.2. TPF Fatty Acid Profiles

Fatty acid methyl esters (FAMEs) were prepared according to Regulation (EU) 2022/2105 [26]. In a screw-top test tube, 0.1 g of the extracted oil was mixed with 2 mL of heptane. The methanolic solution of potassium hydroxide (2 M; 0.2 mL) was then added, and the cap fitted with a PTFE joint was screwed on and shaken vigorously for 30 s. After stratification, the upper phase became clear and contained the FAMEs. The separation of FAMEs was carried out using a Shimadzu gas chromatograph (Shimadzu GC-17A) (Shimadzu Italia, Milan, Italy) equipped with a flame ionization detector (FID) and a capillary column (DB-wax; Agilent Technologies, Wilmington, DE, USA; 30 m length × 0.25 mm i.d. and 0.25 μm of film thickness). As a carrier gas, helium was used (a flow rate of 1.5 mL/min). The initial oven temperature was kept at 165 °C for 15 min and then programmed to rise at 5 °C/min up to 200 °C, maintained for 2 min, and followed by a second gradient of 5 °C/min to a final temperature of 260 °C, which was held for 5 min. The injector and detector temperatures were 260 and 300 °C, respectively. Hydrogen and compressed air were used for the flame detector. The injection volume was 1 μL with a split ratio of 50. Identification of each fatty acid was performed by a comparison of the retention times of the corresponding peak with that of reference standards. The results were expressed as percentages of total fatty acids.

#### 2.1.3. Extraction of TPF Polyphenols

An aliquot (about 150 g) of TPF described above was suspended in 400 mL of three different solvents/solvent mixtures: distilled water (W); distilled water–ethanol 75:25 *v*/*v* (WE), and distilled water–ethanol–formic acid 70:29:1 *v*/*v* (WEF). Extractions were carried out overnight at 4 °C in the dark; after that, they were filtered using a Whatman^®^ qualitative filter paper, Grade 1 (Merck KGaA, Darmstadt, Germany), with the help of a vacuum pump. After removing the alcohol present in WE and WEF samples at reduced pressures (Rotavapor^®^, BUCHI Italia srl, Cornaredo, Italy), the resulting aqueous solutions (WE, WEF, and W) were frozen at −20 °C and then lyophilized with a yield of 3.45 ± 0.07%.

#### 2.1.4. Total Polyphenol Content and Antioxidant Activity of TPF Extracts

TPF extracts (W, WE, WEF) obtained as described in the previous paragraph were characterized for total polyphenol content (TPC) and antioxidant activity (AA). An aliquot of the lyophilized samples (1 g) was resuspended in distilled water (8 mL) and filtered through 0.45 μm Albet filters (Hahnemühle FineArt GmbH, Dassel, Germany). The TPC of W, WE, and WEF TPF extracts was evaluated following the assay of Folin–Ciocolateau (FC) [27] gallic acid as a standard. An aliquot of each extract (100 µL) was mixed with the FC reagent (750 μL; 1:10) and allowed to react for 5 min in the dark; then, 750 μL of Na_2_CO_3_ (60 g/L, *w*/*vol*) was added, and the mixture was kept in the dark for 90 min. The absorbance was measured at 725 nm (Perkin Elmer lambda 25 UV-VIS spectrometer), and the results were expressed as µg of gallic acid equivalents (GAE)/g of lyophilized tomato extract.

The antioxidant capacity of the TPF extracts, resuspended in distilled water, was determined by the DPPH assay, following the method reported by Brand-Williams et al. [28], with some modifications. An aliquot (3 mL) of the DPPH solution (3.94 mg/mL of methanol) was mixed with 50 µL of the extract, homogenized, and incubated in the dark for 60 min at 25 °C.

After incubation, the absorbance of each sample was spectrophotometrically read at 515 nm, using a Perkin Elmer lambda 25 UV-VIS spectrometer. The radical scavenging activity percentage (RSA%) was calculated according to the following formula:

RSA%: [(Absorbance blank − Absorbance sample)/Absorbance blank] × 100(1)

#### 2.1.5. HPLC/DAD and HPLC/ESI/MS Analyses of TPF Extracts

Variable aliquots of the freeze-dried TPF extracts (see Section 2.1.4) were redissolved in 80% aqueous methanol, further filtered, and sent for analytical determinations. HPLC/DAD analyses were carried out on an Ultimate3000 “UHPLC focussed” instrument equipped with a binary high-pressure pump, a Photodiode Array detector, a Thermostatted Column Compartment and an Automated Sample Injector (Thermo Fisher Scientific, Waltham, MA, USA). Collected data were processed through a Chromeleon Chromatography Information Management System v. 6.80. Chromatographic runs were all performed using a method developed by Siracusa et al. [29]. A series of HPLC/ESI/MS analyses were also performed on the extracts to unambiguously identify chromatographic peaks; the HPLC apparatus used was the same as described above, whilst ESI mass spectra were acquired by a Thermo Scientific Exactive Plus Orbitrap MS (Thermo Fisher Scientific, Waltham, MA, USA), using a heated electrospray ionization (HESI II) interface (Thermo Fisher Scientific, Waltham, MA, USA). Mass spectra were recorded operating in positive and negative ion mode in the m/z range of 120–1500 at a resolving power of 25,000 (full width at half maximum, at *m*/*z* 200, RFWHM), resulting in a scan rate of >1.5 scans/s when using an automatic gain control target of 1.0 × 106 and a C-trap inject time of 250 ms. under the following conditions: capillary temperature 300 °C; nebulizer gas (nitrogen) with a flow rate of 60 arbitrary units; auxiliary gas flow rate of 10 arbitrary units; source voltage 3 kV; capillary voltage 82.5 V; and tube lens voltage 85 V. The Orbitrap MS system was tuned and calibrated in positive modes, by infusion of solutions of a standard mixture of sodium dodecyl sulphate (Mr 265.17 Da), sodium taurocholate (Mr 514.42 Da), and Ultramark (Mr 1621 Da). Data acquisition and analyses were performed using the Xcalibur 7.0 ^TM^ software. The results are reported in micrograms (μg) of compound per gram (g) of extract.

### 2.2. Preparation of Functional Bread Samples Using TPF as Partial Wheat Flour Substitute

Bread samples were prepared with durum wheat semolina, TPF (10 and 20%, *w*/*w*), tap water, compressed yeast, NaCl, and extra virgin olive oil (EVO), following the experimental scheme reported in Table 1. All the ingredients apart from TPF were bought in a local supermarket.

The doughs were leavened and baked in a bread maker machine (Moulinex OW240E, Écully, France). Bread samples were left to cool up to 20 °C, and the colour parameters of the loaf were determined; the samples were then grounded in a home grinder (La Moulinette, Moulinex, Écully, France) before chemical analyses. Two independent baking tests were run, and all the analyses were performed in triplicate. Thus, the reported result of each analytical determination is the average of 6 values (2 sample replicates × 3 analytical replicates) unless otherwise stated.

#### 2.2.1. Physico-Chemical Analyses of Functional Bread Samples

Moisture content was determined by gravimetric analysis using an oven (ThermoScientific, Herathermoven, Italy) at 105 °C for 4 h. Total carotenoid content was determined according to the methodology reported in Section 2.1.1. pH values and the total titratable acidity (TTA) were determined, using a pH metre (Mettler Toledo, MP 220, Milano, Italy), as follows: A total of 10 g of the grounded sample was mixed with 90 mL of distilled water. First, the pH value was read, and then the suspension was titrated with 0.1 M NaOH to a pH of 8.5. The TTA was expressed on dry matter as ml of NaOH needed for titration [30].

TPC and AA were determined according to the methods described in Section 2.1.4 on WE extract obtained as follows: 1 g of grounded bread sample was added with 8 mL of WE, and after 10 min of stirring, the solution was centrifuged at 10 °C for 15 min at 8000 rpm (ALC 4128, Cologno Monzese, MI, Italy).

Bread colour evaluation was performed by the colorimeter Konica Minolta CM-2500d (Bremen, Germany), with the illuminant D65. The CIE L*a*b* and C and h were determined on ten different points both on the crust and crumb. For the colour evaluation of the crumb, bread loaves were cut into slices of 2 cm. The colour differences among the control bread and samples with different amounts of TPF were expressed as Δ*E*, calculated using the following equation:

(2) $$\Delta E=\sqrt{{\left(L_{x}-L_{0}\right)}^{2}+{\left(a_{x}-a_{0}\right)}^{2}+{\left(b_{x}-b_{0}\right)}^{2}}$$

where the subscript “*x*” indicates the bread formulated with 10 or 20% TPF, and the subscript “0” indicates the control.

#### 2.2.2. Sensory Evaluation of Functional Bread Samples

The sensory evaluation of bread was carried out using descriptive analysis, performed in the Sensory Laboratory of the Department of Agricoltura, Alimentazione e Ambiente (Di3A), University of Catania (Italy), designed according to the UNI EN ISO 8589: 2014 guidance [31]. The sensory profile of the bread samples was determined according to the UNI EN ISO 13299:2016 method [32] and was carried out by 12 trained panellists with several years of tasting experience, often used in our previous studies on bread. The panellists agreed to take part in the research and signed an informed consent form in accordance with the principles of the Declaration of Helsinki, as our institution does not have an ethics committee for sensory and food quality evaluation studies.

A list of descriptors was generated by the panellists using handmade bread for the sensory evaluation (Table 2) [7,9].

The bread samples were cut into 15 mm thick slices 10 min before tasting. The first and last bread slices were discarded, and the bread slices were served on a plastic plate coded with three-digit numbers. The order of presentation of the samples was balanced using all the presentation combinations possible. Samples were judged without repetition. Judges were provided with water to rinse their mouths between samples. The judges rated the intensity of the selected sensory attributes on a frequency scale from 1 (no sensation/extremely weak) to 9 (extremely intense) (Table 2) (Smart Sensory Solutions S.r.L., Sassari, Italy). Data are expressed as mean ± standard deviation.

### 2.3. Statistical Analysis

A one-way analysis of variance (ANOVA) of experimental data was performed using Minitab19 statistical software (Minitab, State College, PA, USA). When significant differences were assessed by an ANOVA (*p* < 0.05), Fisher’s Least Significant Difference (LSD) test was calculated at the 95% confidence level.

## 3. Results

### 3.1. Physico-Chemical, Compositional, and Functional Characterization of TPF and TPF Extracts

Characterization of TPF was carried out, and the data are presented in Table 3. The TP was dried to a residual moisture of about 3.53%. TPF was characterized by a lipid content of 12.3%, higher than the amount reported by Azabou et al. [11]. The distribution of fatty acids showed that the two most important were linoleic (49.90%) and oleic (20.67%), followed by palmitic (18.45%) and stearic (5.94%) (Table 3). The polyunsaturated fatty acid (PUFA) content of TPF lipid was 52.01% and the polyunsaturated fatty acid/saturated fatty acid (PUFA/SFA) index was 2.01, suggesting a beneficial effect on cardiovascular health [33]. TPF is an important source of dietary fibre with a total fibre content of approximately 52.3%, of which 5.3% was soluble and 47% was insoluble. These values were similar to those reported by Azabou et al. [11], but lower than those reported by Navarro-González et al. [14] due to the genetic diversity and climatic variations of tomato fruits. The insoluble/soluble fibre ratio was about 8.9, as tomato pomace and seeds are usually rich in cellulose, hemicellulose, and lignin [11]. In addition, TPF is a potential source of natural antioxidants and contains remarkable residual levels of both total carotenoids and polyphenols (Table 3).

Data in Table 4 show that both the amount and the distribution of the polyphenols strongly depend on the solvent used. In fact, the water–ethanol–formic acid mixture (WEF) extracted the highest amount of polyphenol, bringing the total amount from 1500 µg/g of total polyphenols in water (W) and water–ethanol systems (WE) to 7900 µg of total polyphenols/g of the TPF extract. The highest antioxidant activity is measured in the WE extract (69.4%) and the lowest is measured in the water one (6.3%). Moreover, these data also show that the antioxidant activity, measured after the DPPH method, is not directly correlated to the total polyphenols, as demonstrated by data related to W and WE systems, showing almost the same level of polyphenols but the widest difference in DPPH value. On the other hand, the WEF extraction system, although showing the highest polyphenol value, had a lower antioxidant activity with respect to the WE system. It was reported that the antioxidant activity determined by DPPH on commercial vegetable juices was poorly correlated (r^2^ = 0.50) with TPC determined by the Folin–Ciocalteau method, as polyphenolic compounds showed different behaviour towards the DPPH free radical, both in terms of capacity and scavenging rate [34].

**Figure 1:**
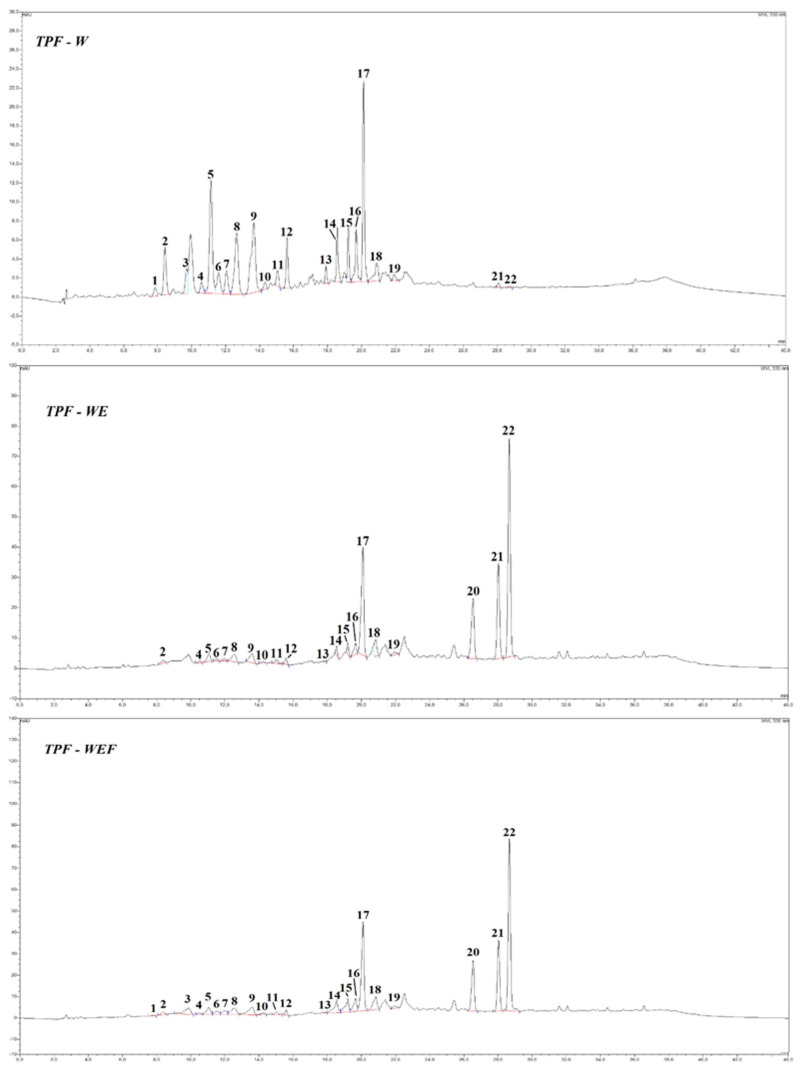
HPLC/DAD profiles, visualized at 330 nm, of tomato pomace flour (TPF) extracts. W = water extract; WE = hydroalcoholic extract; WEF = weakly acidic hydroalcoholic extract. See Table 4 for numbering and text for details.

Figure 1 shows the HPLC profile, visualized at 330 nm, of the TPF extracts. Tomato is indeed widely reported to contain a plethora of specialized metabolites, including flavonoids (flavonols and flavanones), hydroxycinnamic acids, and their derivatives with glucose and/or quinic acid [35]. In the extract object of this study, a total of 22 peaks were detected and tentatively identified, as reported in Table 4. All compounds belong to the biochemical class of polyphenols, and more specifically to the subclasses of hydroxycinnamic acid derivatives (15 peaks) and flavonoids (7 peaks: peak 12, peaks 14–17, peaks 21–22). Among the possible hydroxycinnamic acid derivatives, those with quinic acid and a hexose (presumably glucose) clearly dominate the chromatogram; the preferred hydroxycinnamic acid was undoubtedly caffeic acid (seveb different derivatives), followed by p-coumaric and ferulic acid with four derivatives each. In our previous studies, we found that small-sized tomatoes accumulate remarkable amounts of these molecules, especially in long-shelf-life ecotypes [29,36]. Regarding flavonoids, our results fell in line with data in the literature reporting flavonols quercetin and kaempferol, as well as the flavanone naringenin as representative of this subclass [35].

Similarly to what was found for TPC and antioxidant activity values, the extracts are characterized by different profiles depending on the extraction solvent used.

In particular, the presence of ethanol in the extraction solvent mixture (samples WE and WEF) apparently promotes the extraction of flavonoid aglycones (naringenin and quercetin), whilst water used as the sole solvent (sample W) preferably brings into the solution the hydroxycinnamic acid derivatives (Figure 1 and Table 4). This phenomenon is broadly known in the literature, as also reported by our group for a similar matrix [37].

### 3.2. Characterization of Bread with TPF

The results presented in Table 5 and Table 6 suggest that the addition of TPF to the bread formulation affects several properties. During the baking process, water evaporation affects the weight of the bread, and the addition of TPF increases the weight of the bread samples, suggesting that TPF can reduce the baking loss, probably due to the high fibre content of tomato peels, which binds more water [38]. The height of the bread is consistent within the samples, indicating that the addition of TPF does not significantly affect this parameter (Table 5). The addition of tomato pomace seeds up to 10% resulted in increased loaf volume, porosity, and elasticity. However, higher levels of addition led to a decline in these physical characteristics, likely due to the gluten dilution effect [21]. Conversely, the addition of 6% tomato waste (including seeds and skins) resulted in a reduction in loaf volume. This may be attributed to a number of factors, including yeast, fermentable sugars, pH, and gas retention, which are influenced by fibre content [39].

**Figure 2:**
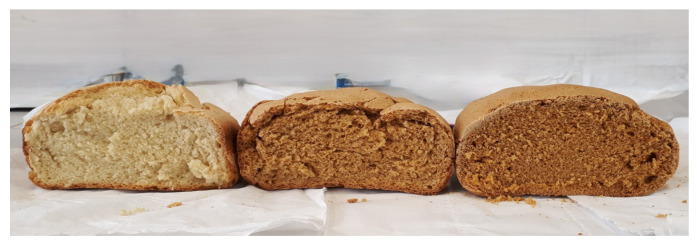
Bread with different levels of tomato pomace; from left to right, control with semolina and bread with 10 and 20% of tomato pomace.

The replacement of semolina with different levels of TPF induced significant differences in the colour parameters both between the fortified bread samples and the control bread (Figure 2). The control bread showed the highest L* value in both crust and crumb, while increasing the percentage of TPF decreased the L* value. The a* and b* parameters showed significant differences between the samples, highlighting the lowest values in the control bread, and increasing the levels of TPF increased the a* and b* parameters (Table 5). A similar trend was observed for the colour of bread, muffins, cream crackers, and cookies containing increasing amounts of tomato pomace: their colour became darker, more red and yellow with the incorporation of tomato pomace [18,19,22]. The ΔE value confirmed the extent of colour changes due to the addition of tomato pomace. In the crumb, the ΔE value was 20.7 ± 2.5 and 29.1 ± 1.6 for 10% and 20% TPF, respectively. The differences between the crusts of the bread samples were limited in relation to the crumb, but visible, with ΔE values of 11.6 ± 1.4 and 18.8 ± 1.8 for 10% and 20% TPF, respectively. Thus, the colour differences between the bread samples were visible to the naked eye.

Table 6 shows the data on moisture, pH, acidity, fibre, carotenoids, total polyphenols, and DPPH activity for the bread samples.

Bread samples with TPF had the highest moisture content, confirming the ability of TPF to reduce water losses during baking. The moisture content in bread supplemented with tomato pomace increased with increasing tomato waste level, due to a greater water-holding capacity of the dry tomato waste than that of the wheat flour [39]. The pH value shows a significant decrease as the percentage of TPF increases. The titratable acidity also increases with the addition of TPF: the 20% TPF bread sample has the highest titratable acidity, while the control has the lowest. Even without considering the contribution of organic acids from fermentation processes to the distinctive aroma of bread, the titratable acidity in bread can have a significant impact on other aspects of the bread flavour profile. The higher the titratable acidity, the tangier the bread will taste. The presence of organic acids can impart a pleasant sour or pungent flavour, enhancing the overall flavour profile of bread. The fibre content of the bread increases as the percentage of TPF increases due to the high fibre level of TPF (Table 3). The sample with 20% TPF has the highest fibre content (about 9.6%). Both fortified TPF breads could use the following nutrition and health claims on the label according to Regulation (EC) 1924/2006: “High Fibre” [40].

The control bread had the lowest level of carotenoids (0.6 mg/kg), while the samples with TPF showed an increased amount of carotenoids, with the highest concentration at 20% TPF (5.0 mg/kg). These data indicate that the contribution to carotenoids is totally and proportionally related to the amount of TPF added. Similarly to the carotenoids, the control has the lowest polyphenol content (292.6 mg/kg) and the TPF-added samples showed higher polyphenol levels, with the highest concentration at 20% TPF (708.9 mg/kg). The DPPH percentage represents the ability of the samples to scavenge DPPH radicals, with a higher percentage indicating greater antioxidant activity. The TPF bread samples showed the highest antioxidant activity, with the highest DPPH percentage observed in the bread sample with 20% TPF (16.9%). The use of TPF increases the levels of carotenoids and polyphenols and potentially enhances antioxidant activity.

In this paper, for the first time, the sensory profile of the bread with TPF was determined. Table 7 shows the mean intensity values perceived in the different bread samples for the sensory attributes of appearance, odour, flavour, taste, and texture in the different bread samples.

As already suggested by the colour parameter results, the bread sample with 20% TPF had the highest crumb colour intensity due to the presence of TPF rich in carotenoids. Regarding the uniformity of alveolation, no differences were observed between the control bread and the bread with TPF, but the samples with 10% TPF had the most consistent and uniform alveolation.

Increasing the level of TPF in bread decreased the typical odour of bread and increased the odour of fresh and dried tomatoes. Both attributes were perceived in bread with TPF with a mean intensity that increased as the percentage of TPF increased. The addition of 20% TPF increased the intensity of the dried tomatoes’ odour more than that of fresh tomatoes.

Among the flavour attributes, “bread” and “yeasty” were perceived less as the TPF content increased. On the contrary, the dried tomato flavour showed a significant increase as the percentage of TPF in the bread formulation increased, suggesting that the use of TPF influenced the typical taste and odour of this baked product. The fresh tomato flavour attribute showed more intensity in the bread with TPF, but this attribute seems to be less influenced by the levels of TPF in the bread. The intensity of sweet, salty, bitter, and astringency was similar between the bread samples. The intensity of sourness increased significantly only in bread with 20% TPF, in accordance with the trend observed for titratable acidity (Table 6). Regarding textural attributes, the use of TPF significantly increases the perceived surface moistness confirming a greater capacity of TPF to retain water during the cooking process. On the contrary, the use of TPF makes the bread less soft and obviously increases the intensity of the grittiness due to the high fibre content. Finally, the “overall” attribute shows similar values in the three experimental bread samples, suggesting that the overall quality of the bread samples remains relatively constant, regardless of the percentage of TPF.

A sensory acceptability degree was reported for some attributes of bread with tomato pomace. Bread with tomato pomace was found to be more appealing in terms of colour, but the texture was less acceptable, as well as taste and flavour [18]. Furthermore, in contrast with our results, when the TP substitution was increased to 10%, the bread was the least liked [39]. This suggests that tomato pomace from cherry and date tomatoes have a limited impact both on overall acceptability and on some sensory properties of bread with TPF.

## 4. Conclusions

In this study, for the first time, the use of tomato pomace flour (TPF), derived from the processing of cherry and date tomatoes, as an ingredient to improve the nutritional value of durum wheat bread was proposed. TPF is a rich source of polyunsaturated and monounsaturated fatty acids, dietary fibre, and polyphenols, and its use, even at a level of 10% in the formulation of durum wheat bread, allows for the production of bread high in fibre with a considerable content of bioactive compounds. In addition, the use of TPF reduces the weight loss of the bread during baking, allowing it to produce a product with a maximum moisture content in accordance with current legislation. This technological advantage could encourage the food industry to use this low-cost waste.

From a sensory point of view, TPF-fortified breads showed an overall acceptance similar to that of the control bread, although the addition of TPF increases the intensity of the typical tomato colour, flavour, and odour as well as the acidity. No effect seems to be induced on texture characteristics such as softness, cohesiveness, dryness, and chewiness, which could negatively affect consumer acceptance.

Furthermore, the proposed use of TP is in line with the EU’s “Farm to Fork” strategy, both from a health and environmental point of view, providing an opportunity for the food industry to adopt sustainable practices and stimulate product reformulation to improve nutrient profiles using production waste.

## Figures and Tables

**Table 1:** Formulation of durum wheat semolina and TPF in dough samples.

Sample Code	Durum Wheat Semolina (g)	TPF (g)	Water (g)	Compressed Yeast (g)	NaCl (g)	EVO (g)
Control	500	−	350	7	12	10
TPB 1	450	50	350	7	12	10
TPB 2	400	100	350	7	12	10

**Table 2:** Sensory attributes, definitions, and anchors used in the descriptive analysis of bread samples.

Attribute	Definition	Scale Anchors	
		1	9
**Visual appearance**			
Crumb colour	Strength of colour from light to dark	Light yellow	Brown
Alveolation uniformity (crumb)	Porosity and homogeneity of the size of the holes	Fine and very homogeneous	Heterogeneous
**Odour/flavours**			
Bread	Intensity of the characteristic odour/flavour of freshly baked bread	Extremely weak	Extremely intense
Yeasty (crumb)	Intensity of the characteristic odour/flavour associated with yeast used as a leaving agent	Extremely weak	Extremely intense
Fresh tomato	Intensity of the characteristic odour/flavour associated with tomato	Extremely weak	Extremely intense
Dried tomato	Intensity of the characteristic odour/flavour of dried tomato	Extremely weak	Extremely intense
Off-odour/off-flavour	Odour/flavour unpleasant, not characteristic of bread	Extremely weak	Extremely intense
**Taste**			
Sweet	Primary sensation produced by sugars	Extremely weak	Extremely intense
Salty	Primary sensation produced by sodium chloride	Extremely weak	Extremely intense
Sour	Primary sensation produced by citric acid	Extremely weak	Extremely intense
Bitter	Primary sensation produced by caffeine	Extremely weak	Extremely intense
Astringency	Sensations of shrinking, puckering, or roughing in the mouth	Extremely weak	Extremely intense
**Texture**			
Surface moistness	Degree of moistness perceived on the surface of the product when in contact with the lips	Dry	Wet
Softness	Degree of softness in the mouth	Soft	Hard
Cohesiveness	Degree to which the chewed sample holds together	Extremely weak	Extremely strong (cohesive mass)
Dryness	Degree of drying effect, amount of saliva absorbed by the sample during chewing	Extremely weak	Extremely strong
Coarse/grittiness	Degree of the presence of small insoluble particles in the mouth after ingesting the sample	Extremely weak	Extremely strong
Chewiness	Number of chews required before swallowing	Few (N ≤ 3)	Several (N ≥ 7)
Overall acceptance	Degree of overall acceptance considering all attributes	Low	High

**Table 3:** Chemical characterization of TPF.

Parameters	
Moisture (%)	3.53 ± 0.85
Lipid (%)	12.3 ± 0.66
Total fibre (%)	52.3 ± 0.65
Soluble fibre (%)	5.3 ± 0.5
Insoluble fibre (%)	47 ± 0.58
Carotenoids (mg/kg)	30.9 ± 4.82
**Fatty Acids (%) ^a^**	
Lauric acid (C12:0)	<0.1
Myristic acid (C14:0)	0.48
Palmitic acid (C16:0)	18.45
Palmitoleic acid (C16:1	0.28
Heptadecanoic acid(C17:0)	0.18
Stearic acid (C18:0)	5.94
Oleic acid (C18:1n-9)	20.67
Linoleic acid (C18:2n-2)	49.90
Arachidic acid (C20:0)	0.52
cis−11Eicosenoic acid (C20:1n-11)	0.13
Linolenic (C18:3n-3)	2.11

^a^ Standard deviation does not exceed 5%.

**Table 4:** Total polyphenols (spectrophotometric), antioxidant activity, and compositional data (reported in μg/g matrix) of TPF extracts.

		W	WE	WEF
Spectrophotometric				
	Total Polyphenols (µg GAE/g)	1488.4 ± 62.95c	1599.4 ± 30.12b	7913.7 ± 112.3a
	Antioxidant Activity (DPPH %)	6.3 ± 0.22c	69.4 ± 0.12a	51.4 ± 0.08b
**Individual compounds 1–22, from HPLC-DAD/MS (µg/g)**				
1	p-coumaroyl hexose isomer 1	0.77 ± 0.12a	n.d.	0.53 ± 0.01ab
2	p-coumaroyl hexose isomer 2	4.39 ± 0.27a	1.20 ± 0.19b	0.79 ± 0.20bc
3	p-coumaroyl hexose isomer 3	2.18 ± 0.12a	n.d	0.16 ± 0.01b
4	caffeoyl hexose isomer 1	1.07 ± 0.07b	0.58 ± 0.02c	1.56 ± 0.01a
5	p-coumaroyl hexose isomer 4	11.76 ± 0.64a	2.94 ± 0.55b	2.79 ± 0.11b
6	caffeoyl hexose isomer 2	2.35 ± 0.53a	0.84 ± 0.06b	0.76 ± 0.20b
7	caffeoyl hexose isomer 3	2.40 ± 0.23a	0.87 ± 0.04b	1.84 ± 0.10ab
8	caffeoyl hexose isomer 4	9.00 ± 0.41a	3.87 ± 0.44c	4.93 ± 0.16a
9	neo chlorogenic acid	12.68 ± 0.55a	4.56 ± 0.58bc	6.15 ± 0.81b
10	caffeoyl quinic acid isomer 2	0.75 ± 0.04b	0.61 ± 0.07b	1.29 ± 0.18a
11	chlorogenic acid	1.56 ± 0.08a	1.58 ± 0.29a	0.67 ± 0.37ab
12	quercetin di-hexoside derivative	93.18 ± 6.03a	41.54 ± 3.21b	53.20 ± 7.52b
13	feruloyl quinic acid 1	1.31 ± 0.09c	1.59 ± 0.01b	3.35 ± 0.07a
14	quercetin derivative	125.17 ± 5.59c	130.47 ± 7.37b	203.90 ± 20.24a
15	rutin-*O*-pentoside	21.56 ± 0.60a	10.49 ± 2.73c	19.10 ± 1.26b
16	kaempferol di-*O*-hexoside	99.72 ± 2.22b	92.27 ± 16.33b	141.96 ± 22.41a
17	rutin	93.40 ± 4.64b	151.72 ± 21.84a	146.28 ± 27.52a
18	feruloyl quinic acid 2	3.01 ± 0.10b	5.73 ± 0.34a	5.88 ± 0.97a
19	feruloyl quinic acid 3	1.02 ± 0.04b	1.19 ± 0.11b	4.54 ± 0.04a
20	ferulic acid derivative	n.d	12.06 ± 0.90a	6.85 ± 2.12b
21	naringenin	8.29 ± 1.86b	410.78 ± 25.99a	8.29 ± 1.86b
22	quercetin	1.29 ± 0.39c	1510.48 ± 83.80a	1478.98 ± 195.64a

W = distilled water extract; WE = distilled water–ethanol extract; WEF = distilled water–ethanol–formic acid extract. Each value is presented as the mean ± standard deviation of the analytical triplicates; different letters in the same row indicate significant differences (*p* ≤ 0.05). n.d. = not detected.

**Table 5:** Physical properties of bread samples.

Sample	Weight (g)	Height of Bread (cm)	Colour Parameters					
			Crumb			Crust		
			L*	a*	b*	L*	a*	b*
Control	341.7 ± 0.22b	5.9 ± 0.14a	73.1 ± 3.07a	−2.0 ± 1.04c	22.4 ± 1.14c	72.0 ± 2.1a	5.1 ± 2.05c	30.5 ± 1.60c
10% TPF	355.1 ± 4.12ab	6.3 ± 0.57ab	59.4 ± 1.81b	6.7 ± 1.28b	36.8 ± 1.02b	63.9 ± 1.51b	9.2 ± 1.50b	37.7 ± 2.24b
20% TPF	358.9 ± 2.00a	5.9 ± 0.14a	53.0 ± 1.38c	10.5 ± 0.95a	39.4 ± 1.39a	57.4 ± 3.34c	12.3 ± 3.12a	40.0 ± 2.63a

The results are presented as mean ± standard deviation. Different letters indicate significant differences between the samples in the same column.

**Table 6:** Carotenoids, total polyphenols, and antioxidant properties of bread samples.

Sample	Moisture (%)	pH	Acidity ^a^	Fibre (%)	Carotenoids (mg/kg)	Total Polyphenols (mg/kg)	DPPH (%)
Control	31.5 ± 0.52b	6.2 ± 0.04a	3.5 ± 0.16c	2.9 ± 0.12c	0.6 ± 0.13c	292.6 ± 45.67c	6.8 ± 4.12c
10% TPF	33.7 ± 0.83a	5.7 ± 0.02b	5.2 ± 0.29b	6.2 ± 0.24b	2.4 ± 0.28b	552.8 ± 92.48b	12.4 ± 4.85b
20% TPF	33.9 ± 0.85a	5.4 ± 0.01c	6.9 ± 0.24a	9.6 ± 0.28a	5.0 ± 0.57a	708.9 ± 106.69a	16.9 ± 6.25a

^a^ mL of NaOH 0.1N. The results are presented as mean ± standard deviation. Different letters indicate significant differences between the samples in the same column.

**Table 7:** Sensory analysis of visual, odour, taste, and texture attributes of bread samples.

Attribute	Bread Samples		
	Control	10%TPF	20%TPF
**Visual appearance**			
Crumb colour	1.36 ± 0.50c	5.21 ± 0.98b	6.79 ± 0.70a
Alveolation uniformity	4.07 ± 1.07ab	4.93 ± 0.92a	3.93 ± 0.92b
**Odour**			
Bread	5.64 ± 0.84a	4.64 ± 0.75b	3.86 ± 0.95c
Yeasty	3.79 ± 0.70a	3.50 ± 0.86a	3.57 ± 0.85a
Fresh tomato	1.07 ± 0.27b	2.64 ± 0.93a	2.71 ± 0.73a
Dried tomato	1.00 ± 0.00c	2.86 ± 0.77b	4.21 ± 0.80a
Off-odour	1.14 ± 0.36a	1.29 ± 0.61a	1.57 ± 0.85a
**Flavour**			
Bread	6.07 ± 0.99a	4.64 ± 0.84b	3.79 ± 0.89c
Yeasty	3.79 ± 0.80a	2.21 ± 0.70b	2.14 ± 0.86b
Fresh tomato	1.64 ± 1.33b	2.36 ± 0.93ab	2.79 ± 0.89a
Dried tomato	1.14 ± 0.36c	3.79 ± 0.80b	5.14 ± 0.77a
Off-flavour	1.21 ± 0.43a	1.29 ± 0.61a	1.57 ± 0.85a
**Taste**			
Sweet	3.00 ± 0.96a	2.79 ± 0.98a	2.71 ± 0.99a
Salty	3.93 ± 0.83a	3.64 ± 0.93a	3.57 ± 0.76a
Sour	1.64 ± 0.93b	1.93 ± 0.82b	3.43 ± 0.85a
Bitter	1.21 ± 0.43a	1.36 ± 0.63a	1.86 ± 0.95a
Astringency	1.43 ± 0.76a	1.64 ± 0.93a	2.21 ± 0.98a
**Texture**			
Surface moistness	4.43 ± 1.02b	5.43 ± 0.94a	5.36 ± 1.01a
Softness	3.64 ± 1.08b	4.57 ± 0.94a	5.29 ± 0.99a
Cohesiveness	5.14 ± 0.95a	5.21 ± 1.12a	5.14 ± 0.77a
Dryness	3.29 ± 0.99a	3.14 ± 0.95a	3.14 ± 0.66a
Coarse/grittiness	2.29 ± 0.61c	3.43 ± 0.94b	4.64 ± 0.93a
Chewiness	5.07 ± 1.07a	4.57 ± 1.09a	4.86 ± 0.95a
Overall acceptance	7.00 ± 1.04a	6.57 ± 1.01a	6.71 ± 1.38a

For each descriptor, different letters in a row denote significant differences between bread samples according to the Fisher LSD multiple comparison test (*p* < 0.05).

## Data Availability

The original contributions presented in this study are included in the article. Further inquiries can be directed to the corresponding author.
